# Sagittal spine disposition and pelvic tilt during outdoor fitness equipment use and their associations with kinanthropometry proportions in middle-aged and older adults

**DOI:** 10.7717/peerj.12657

**Published:** 2021-12-20

**Authors:** Tomás Abelleira-Lamela, Raquel Vaquero-Cristóbal, Noelia González-Gálvez, Francisco Esparza-Ros, Alejandro Espeso-García, Pablo Jorge Marcos-Pardo

**Affiliations:** 1 Injury Prevention in Sport Research Group. Faculty of Sport Science, Catholic University San Antonio of Murcia, Murcia, Spain; 2 Injury Prevention in Sport Research Group. International Chair of Kinanthropometry, Catholic University San Antonio of Murcia, Murcia, Spain; 3 SPORT Research Group (CTS-1024). Department of Education, Faculty of Education Sciences, University of Almería, Almería, Spain; 4 Active Aging, Exercise and Health/HEALTHY-AGE Network, Consejo Superior de Deportes (CSD), Ministry of Culture and Sport of Spain, Madrid, Spain

**Keywords:** Ergonomic, Spinal curvature, Posture, Public facility, Sports and recreational facilities, Sports equipment

## Abstract

**Background:**

Outdoor fitness training has become popular as a tool for improving the health, especially middle-aged and older adults. For this purpose, outdoor fitness equipment (OFE) have been installed in public areas. However, their safety and effectiveness are still unknown. The aim of the present research was to analyze the sagittal disposition of the spine and pelvic tilt during the use of OFE, and to determine the influence of anthropometric variables on these factors in middle-aged and older adults.

**Methods:**

Seventy healthy volunteers, 56 women and 14 men (age: 63.14 ± 8.19 years) participated in the study. Sagittal spine disposition and pelvic tilt were measured using a Spinal Mouse^®^, in the relaxed standing position, and during the use of the OFE. In addition, kinanthropometry variables were also measured according to the guidelines of the International Society for the Advancement of Kinanthropometry.

**Results:**

Regarding thoracic kyphosis, a significant decrease was found in thoracic kyphosis in the initial position (IP) in single bonny rider (SBR) (*p* = 0.006) and row (*p* = 0.046), and a significant increase in the final position (FP) in the row (*p* = 0.011), surfboard (*p* < 0.001) and air walker (*p* = 0.027) machines. In relation to the lumbar curvature and pelvic tilt, a significant decrease in lumbar lordosis and a decrease in pelvic anteversion were observed in the IP and FP in SBR and row; and in the bike (*p* < 0.001) machine. In the surfboard machine, a significant decrease in lumbar lordosis was found (*p* = 0.002), with no changes in pelvic tilt. According to the multiple linear regression analysis, the subjects with a higher cormic index and height were more at risk of increasing their thoracic kyphosis, decreasing lumbar lordosis and/or decreasing pelvic anteversion towards pelvic retroversion.

**Conclusions:**

Middle-aged and older adults show spinal misalignments when using the OFE with respect to the standing position, showing a decrease in the thoracic kyphosis in IP of SBR and ROW, and a significant increase in the surfboard and air walker, and in the FP of Row, in the lumbar lordosis in all the OFE in sitting and some in standing, and in the pelvic anteversion in all the OFE in sitting. The variables height and the cormic index explained most of the changes in sagittal spine disposition.

## Introduction

Ergonomics is understood as the part of biomechanics that works on the adaptation of machinery and workplaces to optimize them for the safety and efficiency of users (Georgoulas, Linner & Bock, 2014; Signorile et al., 2017). For this purpose, machines and furniture are adapted to the body proportions of the subjects for whom they are destined (Castellucci et al., 2015; Colvin, Flores & Hardi, 2013). Among these machines, we find the ones used for guided bodybuilding exercises, which are very common in fitness centers (Cardozo et al., 2019; Ribeiro, Nunes & Schoenfeld, 2020; Signorile et al., 2017). These strength training machines are designed to be effective and safe for users (Chow, 2013; Chow & Wu, 2019; Coratella et al., 2020). In this way, the machinery is designed so that during sports practice the position of the body is optimal for the desired muscular action, without causing alterations in the rest of the body structures or in the work itself (Coratella et al., 2020; Maffiuletti & Lepers, 2003; Rodriguez-Ridao et al., 2020).

A lack of ergonomics or improper use of weight training machines during training can be detrimental to the health of the person performing the exercise, and can also cause changes in the maximum strength-producing capacity of the user by modifying biomechanics. More specifically, the execution of exercises with an excessive range of motion and the adoption of inadequate postures, especially with regard to the spine, or an incorrect execution of the exercise performed (Chow, 2013; Rodriguez-Ridao et al., 2020), together with an increase in the loads during execution can lead to multiple injuries of different kinds (Hartmann, Wirth & Klusemann, 2013; McGill, Cannon & Andersen, 2014; Sayers et al., 2020). Among the most frequent injuries that could result from overloading caused by incorrect use of this type of machinery we find herniated discs, spondylolysis, and spondylolisthesis (Harrison et al., 2005). However, the origin of injuries and pathologies of both the spine and the adjacent muscular system is multifactorial, so other factors such as technique, fatigue, sarcopenia or age must also be taken into consideration (Gottschlich & Young, 2011; Montero-Fernandez & Serra-Rexach, 2013; Ohyama et al., 2019).

To avoid these situations of lack of ergonomics or improper use of weight training machines during training, as well as the most frequent related injuries (Ailon et al., 2015; Harrison et al., 2005), the vast majority of weight training machines on the market today are adjustable. In this way, they can be adapted to each user, regardless of their anthropometric characteristics and proportions, while maintaining efficiency and safety when training with them. In addition, as they are mostly guided machines, the muscles involved can be worked in a controlled range of motion (Ribeiro, Nunes & Schoenfeld, 2020), which reduces the risk of compensations in other structures. This type of compensation occurs especially in frail subjects with postural deviations or previous spinal injuries, as is the case in middle-aged and older adults (Ribeiro, Nunes & Schoenfeld, 2020). More specifically, previous studies have found that the older population tends to show thoracic hyperkyphosis, reductions in lumbar curvature and intervertebral disc degeneration (Ailon et al., 2015; Ohyama et al., 2019). Recent systematic reviews (Bayattork et al., 2020) and systematic reviews with meta-analysis (Gonzalez-Galvez, Gea-Garcia & Marcos-Pardo, 2019) have proven that systematic strength training can lead to improvement in the sagittal spinal curvatures, or correct malalignments, therefore, it would be a highly recommended training in this population (Ohyama et al., 2019; Toprak-Çelenay & Özer Kaya, 2017).

Despite this evidence, most studies that have analyzed the adaptations that occur in the sagittal spine disposition with the systematic practice of exercise have focused on specific sports in athletes of different categories (Grabara, 2018; López-Miñarro et al., 2017) or certain types of physical exercise in older (Grabara & Szopa, 2015). However, few studies have analyzed the acute influence of physical exercise on the sagittal spine disposition during the use of specific sports equipment. More specifically, the sagittal spine disposition has been analyzed in kayakers or canoeists who were paddling in the boat (Abelleira-Lamela et al., 2020; López-Miñarro, Muyor & Alacid, 2012). or in cyclists when they were standing on the bike (Muyor, 2015; Muyor, López-Miñarro & Alacid, 2011). However, there are no studies that have analyzed the adaptations that occur in the sagittal spine disposition during the use of sports fitness equipment such as gym equipment.

Due to the benefits observed from training with weight training machines such as strength and functional fitness gains, as well as increase in hormones of bone formation in those who use them, especially in older adults (Cardozo et al., 2019; Moghadasi & Siavashpour, 2013), the installation and use of outdoor fitness equipment (OFE) areas as a tool for the improvement of the health of the population, especially middle-aged and older adults, has been popularized by different governmental institutions (Cohen et al., 2012). OFE are outdoor sports facilities commonly located in public places, composed of machines that seek to have a certain similarity with the weight training machines found in indoor sports centers. However, this type of equipment, unlike the equipment used in fitness rooms, is not adjustable, and no studies have been found that have analyzed the sagittal disposition of the spine when using this type of machinery, or the influence that anthropometric proportions could have on them. Therefore, the aim of the present research was to analyze the sagittal disposition of the spine and pelvic tilt during the use of OFE, as well as to determine the influence of anthropometric variables on these factors in middle-aged and older adults. It was hypothesized that there would be changes in sagittal spine disposition and pelvic tilt during the use of OFE and that these changes would depend on anthropometric parameters related to structural bone characteristics in middle-aged and elderly adults.

## Materials & Methods

### Participants

The data used in this cross-sectional study derives from the Intelligent Outdoor Fitness Equipment Project (Design and manufacture of new ergonomic, efficient and healthy outdoor fitness equipment with an application for mobile devices (APP) to assess and control training); code: RTC-2017-6145-1, 2017; with a research grant from the Spanish Ministry of Science, Innovation and Universities.

The sample consisted of 70 healthy volunteers, 56 women and 14 men aged from 50 to 80 years old (mean age: 63.14 ± 8.19 years). The inclusion criteria were: (1) minimum age of 50 years and maximum age of 85 years; (2) functional independence; (3) not being part in a training program during the measurements; (4) not to have any pathology that would prevent the performance of the tests; (5) not having had an operation on the spine or hamstring musculature; and (6) not present a medically diagnosed structural spinal pathology such as a structured thoracic hyperkyphosis, a structured lumbar hyperlordosis, or some kind of structured rectification, etc.

The sample size and power were established taking into account the standard deviation (6.67) of the thoracic curvature variable in a sample with similar characteristics (*n* = 42) (Hasegawa et al., 2018). The sample size of this study was 70 subjects, with a power of 95% and a significance level of 0.05; an estimated error of 1.56° was found. Rstudio 3.15.0 software was used to determine the sample size.

The study was conducted in accordance with the Declaration of Helsinki, and was approved by the institutional ethical committee of the Catholic University of Murcia (Ethical Application Ref: CE111908). This study was designed following the Strobe Statement (Von Elm et al., 2008). All participants were informed about the tests to be performed in this study, and signed an informed consent form before the measurements.

### Measurements

The same trained researcher measured the participants in a single session between 09:00 and 11:00 h. The laboratory temperature was standardized at 24 °C. The participants were instructed to wear lightweight clothes. Participants had not previously exercised on the same day of the assessment and at least two hours had passed since they had been in the supine position (López-Miñarro et al., 2017; Vaquero-Cristóbal et al., 2015).

### Sagittal spine disposition and pelvic tilt

**Figure 1:**
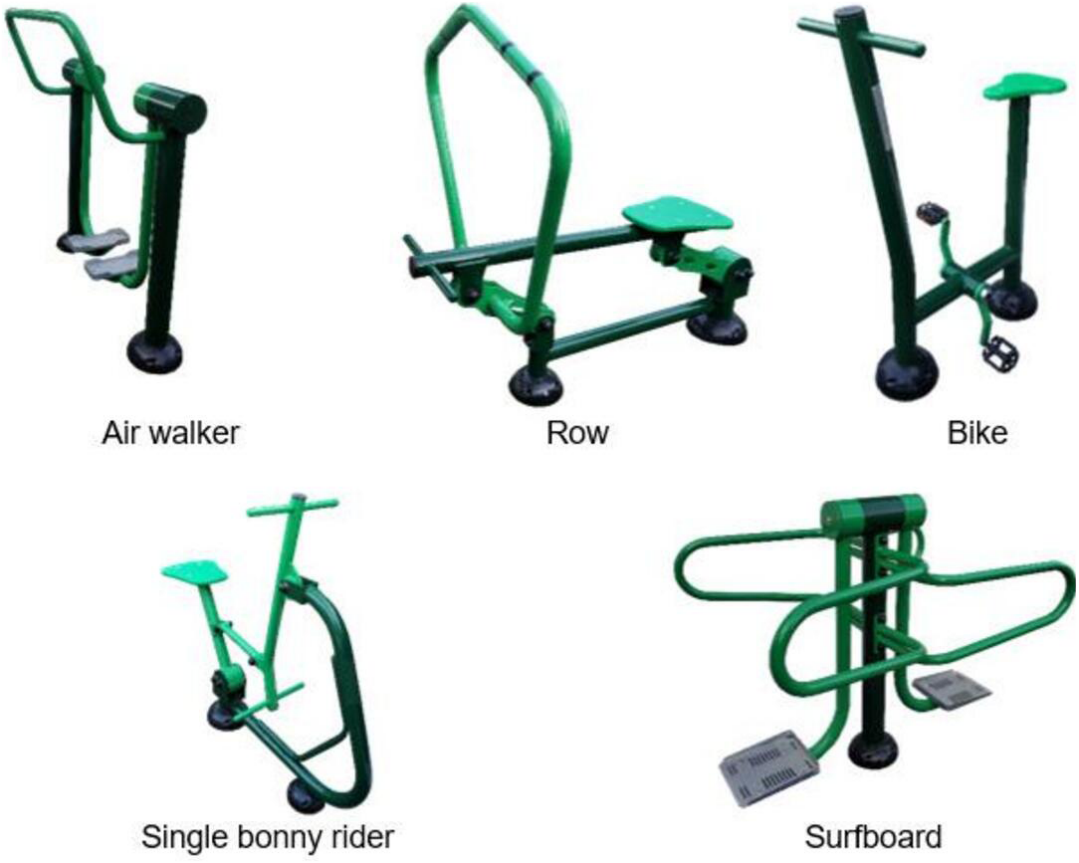
Outdoor fitness equipment.

The sagittal spine disposition and pelvic tilt were measured using the Spinal Mouse^®^ system (Idiag, Fehraltdorf, Switzerland). Measurements were taken randomly in the relaxed standing position, and using the OFE. In the OFE with a cyclic movement, the measurements were taken at the starting position, defining this as the position when the participant was on the OFE, but before starting the movement for which it is intended. In the acyclic OFE, the measurement was made in the same conditions as the cyclic ones, considering this position as the initial position (IP). After that, the subject was asked to execute the movement for which the OFE was designed at a controlled speed until reaching the end of the concentric phase in the OFE. The participant had to maintain this final position (FP) for the sagittal spine disposition to be assessed. Since, the way OFE is used could influence the disposition in it (Chow & Wu, 2019), in the present investigation each OFE was used according to the manufacturer’s recommendations. More specifically, in the air walker the participant did a reverse stride, standing facing forward on the OFE, grasping the front; in the Bike the participant was sitting on the OFE, with one foot on each pedal, hands on the handgrip and crank parallel to the ground; in the surfboard the participant was standing on the platform grasping both sides; in the SBR the participant also was sitting on the seat, with feet on the support, and grasping the handgrip with hand supine, pull with arms to 90° elbow flexion; and in the Row the participant was sitting on the seat, grasping the handgrip with hands about shoulder width apart, and feet on the supports, pull on the handgrip to 90° elbow flexion. The outdoor fitness pieces of equipment were obtained from the company Entorno Urbano S.L.U (Murcia, Spain) (Fig. 1).

Each position was performed twice, and the final value of each measurement was the mean of both measurements. Five minutes were left between the different positions to avoid acute adaptations to the maintenance of a given position in an isometric manner following the methodology of previous studies (López-Miñarro et al., 2017). For thoracic and lumbar curvatures, a positive value indicated that the curve was kyphotic, while a negative value indicated that the curve was lordotic. For pelvic tilt, a positive value indicated that the presence of pelvic anteversion, while a negative value indicated the presence of pelvic retroversion.

### Kinanthropometry

The measurements were taken according to the guidelines of the International Society for the Advancement of Kinanthropometry (Esparza-Ros, Vaquero-Cristóbal & Marfell-Jones, 2019) by an accredited anthropometrist. The measurements taken were body mass, stretch stature, sitting height, waist and hip girths, arm and forearm length, and biacromial and biiliocristal breaths. A Tanita BC-545N scale (Tanita, Arlington Heights, Illinois, USA) was used to measure body mass, and a HR001 portable stadiometer (Tanita, Arlington Heights, Illinois, USA) was used to measure stretch stature and sitting height. The lengths were taken with a Pro model segmometer (Cescorf Ltd., Porto Alegre, Brazil), and breaths with a large sliding caliper (Cescorf Ltd., Porto Alegre, Brazil). Each measurement was taken twice or three times if the difference between the first two measurements was greater than 1%, taking the mean or median, respectively. With the measurements taken, the BMI (weight/(Height(m)^2^)), cormic index ((sitting height/height) × 100), waist-hip ratio (Waist girths/Hip girths); cormic index; upper limb length (arm length + forearm length) and acromio-iliac index ((biiliac diameter/biacromial diameter) × 100) were calculated as previously described in Salces et al. (2007).

### Statistical analyses

The Kolmogorov–Smirnov test and Mauchly’s W-test were used to evaluate the normality and the sphericity of the data. The mean and standard deviation were calculated, and to determine differences between each curvature in standing position and in each OFE, a *t*-test for correlated samples was used. The effect size was calculated by Cohen’s d coefficient. For this purpose, a value > 0.8 was considered a high effect; a value between 0.6 and 0.8 was considered a moderate-high effect; a value between 0.4 and 0.6 was considered a moderate effect; a value between 0.2 and 0.4 was considered a low-moderate effect; and a value < 0.2 was considered a low effect size (Cohen, 1988). An ANCOVA was performed to analyze the influence of sex and height in the comparison of the differences between the position in standing and in the OFE. The effect size was calculated with partial eta squared (*η*_p_^2^). To determine the association between sagittal spine disposition and pelvic tilt during OFE use and kinanthropometry proportions Pearson’s bivariate correlation coefficients were used. The established ranges of correlation were as follows: *r* > 0.7 for a high correlation, 0.5–0.7 for a moderate correlation, and < 0.5 for a low correlation. Stepwise multiple linear regression models were used to predict sagittal spine disposition according to kinanthropometry proportions. In case of a nonlinear multiple regression model provided a best explanation of the variance that the multiple linear regression models, the best model association between the dependent and independent variables was explored with a curvilinear estimation The statistical significance was set at *p* < 0.05. The statistical analyses were carried out using SPSS (Version 22, SPSS Inc., Chicago, IL, USA).

## Results

Table 1 show the mean and standard deviation of the middle-aged and older adults in anthropometric basic measures, BMI, girths, lengths, breadths and proportionality variables, as well as the sagittal spine disposition in standing position, and during the use of the OFE. It was found that the sample presented normal BMI and waist-hip ratio values (29.69 ± 4.27 kg/m^2^ and 0.90 ± 0.09, respectively). With respect to the spine disposition, the thoracic curvature in standing position was 50.10 ± 10.67°, the lumbar curvature was −31.85 ± 9.34°, and the pelvic tilt was 21.32 ± 7.31°. In the OFE, thoracic curvatures were between 46.92 ± 11.51° to 53.79 ± 10.59°; lumbar curvatures were between -31.23 ± 10.20° to 4.82 ± 9.47°; and pelvic tilt were between −4.25 ± 9.59° to 22.42 ± 8.39°. The differences in the sagittal spine disposition and pelvic tilt between the different OFE and the standing position is shown in the Table 2. Regarding thoracic kyphosis, a significant decrease was found in the IP in SBR (*p* = 0.006) and row (*p* = 0.046), and a significant increase in the FP in the row (0.011), surfboard (*p* < 0.001) and air walker (*p* = 0.027) machines, with an effect size from low to moderate. In relation to the lumbar curvature and pelvic tilt, a significant decrease in lumbar lordosis and a decrease in pelvic anteversion were observed in the IP and FP in SBR and row; and in the bike (*p* < 0.001) machine, with an effect size from low to high. In the surfboard machine, a significant decrease in lumbar lordosis was found (*p* = 0.002), with a moderate effect size, with no changes in pelvic tilt.

**Table 1:** Characteristics of the participants.

Anthropometric variables	X ± SD	Spine variables	X ± SD
Body Mass (kg)	74.82 ± 13.69	Standing Thoracic curv. (°)	50.10 ± 10.67
Height (cm)	158.56 ± 9.64	Standing Lumbar curv. (°)	−31.85 ± 9.34
BMI	29.69 ± 4.27	Standing Pelvic tilt (°)	21.32 ± 7.31
Sitting height (cm)	82.68 ± 4.99	SBR IP Thoracic curv. (°)	46,92 ± 11.51
Cormic index	52.35 ± 1.44	SBR IP Lumbar curv. (°)	−2.64 ± 10.71
Waist girth (cm)	93.31 ± 11.40	SBR IP Pelvic tilt (°)	11.22 ± 8.50
Hip girth (cm)	104.60 ± 9.90	SBR FP Thoracic curv. (°)	52.24 ± 10.83
Waist-hip ratio	0.90 ± 0.09	SBR FP Lumbar curv. (°)	−14.59 ± 14.27
Arm length (cm)	31.48 ± 2.27	SBR FP Pelvic tilt (°)	9.73 ± 10.59
Forearm length (cm)	23.44 ± 1.95	Row IP Thoracic curv. (°)	47.63 ± 10.47
Upper limb length (cm)	54.95 ± 4.03	Row IP Lumbar curv. (°)	4.82 ± 9.47
Biacromial breadth (cm)	37.16 ± 1.94	Row IP Pelvic tilt (°)	2.49 ± 7.28
Biiliocristal breadth (cm)	31.03 ± 2.20	Row FP Thoracic curv. (°)	53.71 ± 11.91
Acromio-iliac index	0.84 ± 0.06	Row FP Lumbar curv. (°)	−3.65 ± 14.74
		Row FP Pelvic tilt (°)	−4.25 ± 9.59
		Bike Thoracic curv. (°)	48.02 ± 11.68
		Bike Lumbar curv. (°)	−3.12 ± 11.75
		Bike Pelvic tilt (°)	14.80 ± 9.21
		Surfboard Thoracic curv. (°)	53.79 ± 10.59
		Surfboard Lumbar curv. (°)	−26.49 ± 12.75
		Surfboard Pelvic tilt (°)	22.42 ± 8.39
		Air walker Thoracic curv. (°)	52.33 ± 9.82
		Air walker Lumbar curv. (°)	−31.23 ± 10.20
		Air walker Pelvic tilt (°)	19.92 ± 8.17

**Notes.**

BMI Body mass index curv curvature FP Final position IP Initial position MIS maximal isometric strength SBR Single bonny rider

**Table 2:** Analysis of the differences in the spine sagittal curvatures between the OFE and the standing position.

OFE-Position	Variable	Differences OFE-Standing					Model: Differences OFE-Standing x Sex			Model: Differences OFE-Standing x Height		
		X ± DS	*p*	df	Effect size (*d*)	CI 95%	F	p	*η*_p_^2^	F	p	*η*_p_^2^
SBR IP	Thoracic curv. (°)	−3.18 ± 9.00	.006	64	0.29	−5.47; −0.94	.011	.919	.001	.327	.576	.021
	Lumbar curv. (°)	29.21 ± 12.27	<.001	64	2.91	26.08; 32.26	.909	.352	.043	.066	.800	.003
	Pelvic tilt (°)	−10.13 ± 9.39	<.001	64	1.27	−12.50; −7.76	.225	.638	.005	5.098	.029	.108
SBR FP	Thoracic curv. (°)	2.14 ± 8.95	.053	64	0.20	−0.03; 4.46	.393	.540	.026	.202	.659	.013
	Lumbar curv. (°)	17.25 ± 14.23	<.001	64	1.43	13.63; 20.79	2.165	.157	.098	.008	.928	.000
	Pelvic tilt (°)	−11.52 ± 9.50	<.001	64	1.27	−14.00; −9.25	.002	.966	.000	5.391	.025	.114
Row IP	Thoracic curv. (°)	−1.96 ± 8.77	.046	64	0.23	−4.96; −0.04	.033	.859	.002	.044	.836	.003
	Lumbar curv. (°)	36.67 ± 10.88	<.001	64	3.90	34.44; 39.80	.968	.337	.046	.014	.908	.001
	Pelvic tilt (°)	−18.12 ± 8.37	<.001	64	2.58	−20.68; −16.61	.036	.850	.001	5.179	.028	.110
Row FP	Thoracic curv. (°)	2.76 ± 12.43	.011	64	0.32	0.84; 6.19	.500	.490	.032	.493	.493	.032
	Lumbar curv. (°)	28.19 ± 14.35	<.001	64	2.29	24.30; 31.60	1.727	.204	.079	.522	.479	.025
	Pelvic tilt (°)	−25.50 ± 10.11	<.001	64	3.00	−27.63; −22.64	.067	.796	.002	4.671	.036	.100
Bike	Thoracic curv. (°)	−1.32 ± 11.13	.169	64	0.19	−4.34; 0.77	.203	.659	.013	.004	.952	.000
	Lumbar curv. (°)	28.24 ± 13.19	<.001	64	2.71	25.38; 31.65	2.544	.126	.113	.412	.528	.020
	Pelvic tilt (°)	−6.13 ± 10.79	<.001	64	0.78	−9.08; −3.82	4.821	.034	.103	.511	.479	.012
Surfboard	Thoracic curv. (°)	3.69 ± 7.39	<.001	64	0.35	1.74; 5.44	2.672	.123	.151	5.866	.029	.281
	Lumbar curv. (°)	4.62 ± 11.73	.002	64	0.48	2.16; 8.73	.736	.401	.035	3.511	.076	.149
	Pelvic tilt (°)	1.18 ± 6.05	.166	64	0.14	−0.46; 2.62	.937	.339	.022	.010	.920	.000
Air walker	Thoracic curv. (°)	2.23 ± 7.62	.027	64	0.22	0.26; 4.09	1.757	.205	.105	.790	.388	.050
	Lumbar curv. (°)	0.62 ± 8.65	.558	64	0.06	−1.53; 2.82	.984	.333	.047	.001	.974	.000
	Pelvic tilt (°)	−1.10 ± 6.06	.074	64	0.18	−3.02; 0.14	.827	.368	.019	.751	.391	.018

**Notes.**

OFE outdoor fitness equipment IP Initial position FP Final position curv curvature SBR Single bonny rider

ANCOVA analysis showed that sex was not an influential factor in the changes of sagittal spine disposition in the OFE (F = 002–2.672; *p* = 0.123–0.966), with the exception of pelvic tilt in the Bike (*F* = 4.821; *p* = 0.034). When the covariable height was analyzed, it was found that this covariate significantly influenced pelvic tilt in the OFE performed in sitting (SBR and Row in both IP and FP) (*F* = 4.671–5.391; *p* = 0.025–0.036), and thoracic curvature in the surfboard (*F* = 5.866; *p* = 0.029) (Table 2).

Table 3 shows bivariate correlations between spinal curvatures in the OFE and anthropometric variables. In almost cases anthropometric variables were correlated with the results obtained in the measurements of spinal curvatures. More specifically, body mass, BMI, waist-girth, hip girth, and biiliocristal breadth were correlated with thoracic curvatures during the use of the OFE. Regarding lumbar curvatures, the anthropometric variables with significant correlations were height, hip girth, waist-hip ratio, and acromio-iliac index. However, pelvic tilt was the variables which showed a higher number of correlations with anthropometric variables. Correlations were found between pelvic tilt and height, waist-hip ratio, arm length, forearm length, upper limb length, biacromial breadth, and acromio-iliac index.

**Table 3:** Bivariate correlations between spinal curvatures in the outdoor fitness machines and anthropometric variables.

	**Body mass**	**Height**	**BMI**	**Sitting height**	**Cormic index**	**Waist girth**	**Hip girth**	**Waist-hip ratio**	**Arm length**	**Forearm length**	**Upper limb length**	**Biacromial breadth**	**Biiliocristal breadth**	**Acromio-iliac index**
**Standing Thoracic curv.**	***r* = 0.307; *p* = 0.013**	*r* = 0.111; *p* = 0.377	***r* = 0.316; *p* = 0.010**	*r* = − 0.061; *p* = 0.628	***r* = − 0.367; *p* = 0.003**	***r* = 0.442; *p* = 0.000**	***r* = 0.254; *p* = 0.041**	***r* = 0.277; *p* = 0.026**	***r* = 0.268; *p* = 0.031**	*r* = 0.207; *p* = 0.098	***r* = 0.249; *p* = 0.046**	*r* = 0.148; *p* = 0.238	***r* = 0.340; *p* = 0.006**	*r* = 0.237; *p* = 0.057
**Standing Lumbar curv.**	*r* = − 0.231; *p* = 0.064	*r* = − 0.213; *p* = 0.088	*r* = − 0.139; *p* = 0.270	*r* = − 0.172; *p* = 0.171	*r* = 0.097; *p* = 0.442	*r* = − 0.073; *p* = 0.561	***r* = − 0.251; *p* = 0.044**	*r* = 0.154; *p* = 0.222	*r* = − 0.239; *p* = 0.055	*r* = − 0.172; *p* = 0.170	*r* = − 0.215; *p* = 0.085	*r* = 0.011; *p* = 0.931	*r* = − 0.171; *p* = 0.172	*r* = − 0.178; *p* = 0.157
**Standing Pelvic tilt**	*r* = 0.110; *p* = 0.381	*r* = 0.038; *p* = 0.762	*r* = 0.130; *p* = 0.301	*r* = 0.073; *p* = 0.562	*r* = 0.061; *p* = 0.630	*r* = − 0.057; *p* = 0.653	***r* = 0.257; *p* = 0.039**	***r* = − 0.299; *p* = 0.015**	*r* = 0.031; *p* = 0.804	*r* = − 0.035; *p* = 0.783	*r* = 0.000; *p* = 0.998	*r* = − 0.128; *p* = 0.310	*r* = 0.061; *p* = 0.629	*r* = 0.158; *p* = 0.209
**SBR IP Thoracic curv.**	***r* = 0.298; *p* = 0.016**	*r* = 0.109; *p* = 0.388	***r* = 0.302; *p* = 0.015**	*r* = 0.043; *p* = 0.735	*r* = − 0.149; *p* = 0.236	***r* = 0.425; *p* = 0.000**	***r* = 0.247; *p* = 0.047**	***r* = 0.260; *p* = 0.037**	***r* = 0.256; *p* = 0.039**	*r* = 0.168; *p* = 0.180	*r* = 0.223; *p* = 0.075	*r* = 0.104; *p* = 0.411	***r* = 0.269; *p* = 0.030**	*r* = 0.200; *p* = 0.110
**SBR IP Lumbar curv.**	*r* = − 0.057; *p* = 0.652	*r* = 0.024; *p* = 0.849	*r* = − 0.094; *p* = 0.456	*r* = − 0.088; *p* = 0.485	*r* = − 0.239; *p* = 0.055	*r* = 0.026; *p* = 0.835	***r* = − 0.320; *p* = 0.009**	***r* = 0.327; *p* = 0.008**	*r* = 0.014; *p* = 0.915	*r* = − 0.012; *p* = 0.927	*r* = 0.002; *p* = 0.990	*r* = 0.188; *p* = 0.135	*r* = − 0.172; *p* = 0.171	***r* = − 0.322; *p* = 0.009**
**SBR IP Pelvic tilt**	*r* = − 0.199; *p* = 0.113	***r* = − 0.402; *p* = 0.001**	*r* = 0.089; *p* = 0.480	*r* = − 0.230; *p* = 0.065	***r* = 0.364; *p* = 0.003**	*r* = − 0.194; *p* = 0.122	*r* = 0.232; *p* = 0.063	***r* = − 0.432; *p* = 0.000**	***r* = − 0.412; *p* = 0.001**	***r* = − 0.354; *p* = 0.004**	***r* = − 0.399; *p* = 0.001**	***r* = − 0.477; *p* = 0.000**	*r* = − 0.003; *p* = 0.978	***r* = 0.352; *p* = 0.004**
**SBR FP Thoracic curv.**	*r* = 0.170; *p* = 0.175	*r* = − 0.110; *p* = 0.382	***r* = 0.328; *p* = 0.008**	*r* = − 0.147; *p* = 0.244	*r* = − 0.087; *p* = 0.490	***r* = 0.401; *p* = 0.001**	***r* = 0.259; *p* = 0.037**	*r* = 0.218; *p* = 0.081	*r* = 0.082; *p* = 0.518	*r* = 0.007; *p* = 0.956	*r* = 0.048; *p* = 0.706	*r* = − 0.057; *p* = 0.649	*r* = 0.134; *p* = 0.288	*r* = 0.180; *p* = 0.152
**SBR FP Lumbar curv.**	*r* = 0.024; *p* = 0.851	***r* = 0.274; *p* = 0.027**	*r* = − 0.220; *p* = 0.078	*r* = 0.181; *p* = 0.150	*r* = − 0.194; *p* = 0.121	*r* = − 0.007; *p* = 0.955	***r* = − 0.368; *p* = 0.003**	***r* = 0.331; *p* = 0.007**	*r* = 0.226; *p* = 0.070	***r* = 0.247; *p* = 0.048**	***r* = 0.245; *p* = 0.049**	***r* = 0.311; *p* = 0.012**	*r* = − 0.070; *p* = 0.581	***r* = − 0.305; *p* = 0.013**
**SBR FP Pelvic tilt**	*r* = − 0.122; *p* = 0.335	***r* = − 0.328; *p* = 0.008**	*r* = 0.135; *p* = 0.282	*r* = − 0.242; *p* = 0.052	*r* = 0.178; *p* = 0.156	*r* = − 0.090; *p* = 0.474	*r* = 0.236; *p* = 0.058	***r* = − 0.316; *p* = 0.01**	***r* = − 0.329; *p* = 0.007**	***r* = − 0.297; *p* = 0.016**	***r* = − 0.326; *p* = 0.008**	***r* = − 0.358; *p* = 0.003**	*r* = 0.017; *p* = 0.892	***r* = 0.289; *p* = 0.020**
**Row IP Thoracic curv.**	*r* = 0.209; *p* = 0.095	*r* = 0.055; *p* = 0.664	*r* = 0.234; *p* = 0.061	*r* = 0.039; *p* = 0.756	*r* = − 0.036; *p* = 0.778	***r* = 0.262; *p* = 0.035**	*r* = 0.192; *p* = 0.124	*r* = 0.116; *p* = 0.358	*r* = 0.112; *p* = 0.373	*r* = 0.145; *p* = 0.249	*r* = 0.133; *p* = 0.290	*r* = 0.021; *p* = 0.869	*r* = 0.178; *p* = 0.155	*r* = 0.169; *p* = 0.179
**Row IP Lumbar curv.**	*r* = − 0.179; *p* = 0.153	*r* = 0.132; *p* = 0.296	***r* = − 0.369; *p* = 0.003**	*r* = 0.078; *p* = 0.538	*r* = − 0.104; *p* = 0.410	*r* = − 0.157; *p* = 0.213	***r* = − 0.478; *p* = 0.000**	*r* = 0.242; *p* = 0.053	*r* = 0.065; *p* = 0.608	*r* = 0.013; *p* = 0.919	*r* = 0.042; *p* = 0.742	*r* = 0.113; *p* = 0.370	*r* = − 0.178; *p* = 0.155	***r* = − 0.282; *p* = 0.023**
**Row IP Pelvic tilt**	*r* = − 0.010; *p* = 0.937	***r* = − 0.421; *p* = 0.000**	***r* = 0.364; *p* = 0.003**	***r* = − 0.330; *p* = 0.007**	*r* = 0.182; *p* = 0.148	*r* = 0.030; *p* = 0.811	***r* = 0.455; *p* = 0.000**	***r* = − 0.367; *p* = 0.003**	***r* = − 0.401; *p* = 0.001**	***r* = − 0.357; *p* = 0.003**	***r* = − 0.395; *p* = 0.001**	***r* = − 0.379; *p* = 0.002**	*r* = 0.076; *p* = 0.545	***r* = 0.366; *p* = 0.003**
**Row FP Thoracic curv.**	***r* = 0.371; *p* = 0.002**	*r* = − 0.005; *p* = 0.969	***r* = 0.492; *p* = 0.000**	*r* = − 0.035; *p* = 0.782	*r* = − 0.072; *p* = 0.569	***r* = 0.478; *p* = 0.000**	***r* = 0.485; *p* = 0.000**	*r* = 0.104; *p* = 0.408	*r* = 0.062; *p* = 0.622	*r* = 0.081; *p* = 0.519	*r* = 0.074; *p* = 0.557	*r* = 0.004; *p* = 0.977	***r* = 0.353; *p* = 0.004**	***r* = 0.359; *p* = 0.003**
**Row FP Lumbar curv.**	*r* = − 0.177; *p* = 0.159	***r* = 0.245; *p* = 0.049**	***r* = − 0.451; *p* = 0.000**	*r* = 0.178; *p* = 0.157	*r* = − 0.134; *p* = 0.286	*r* = − 0.136; *p* = 0.280	***r* = − 0.614; *p* = 0.000**	***r* = 0.398; *p* = 0.001**	***r* = 0.256; *p* = 0.039**	*r* = 0.137; *p* = 0.276	*r* = 0.207; *p* = 0.098	*r* = 0.212; *p* = 0.089	***r* = − 0.287; *p* = 0.020**	***r* = − 0.461; *p* = 0.000**
**Row FP Pelvic tilt**	*r* = − 0.012; *p* = 0.922	***r* = − 0.320; *p* = 0.009**	***r* = 0.262; *p* = 0.035**	***r* = − 0.245; *p* = 0.049**	*r* = 0.156; *p* = 0.216	*r* = − 0.018; *p* = 0.884	***r* = 0.323; *p* = 0.009**	***r* = − 0.309; *p* = 0.012**	***r* = − 0.269; *p* = 0.030**	*r* = − 0.209; *p* = 0.094	***r* = − 0.250; *p* = 0.045**	***r* = − 0.337; *p* = 0.006**	*r* = 0.031; *p* = 0.807	***r* = 0.293; *p* = 0.018**
**Bike Thoracic curv.**	***r* = 0.369; *p* = 0.002**	*r* = 0.091; *p* = 0.466	***r* = 0.394; *p* = 0.001**	*r* = 0.089; *p* = 0.477	*r* = − 0.012; *p* = 0.926	***r* = 0.431; *p* = 0.000**	***r* = 0.326; *p* = 0.008**	*r* = 0.195; *p* = 0.116	*r* = 0.206; *p* = 0.098	*r* = 0.146; *p* = 0.241	*r* = 0.186; *p* = 0.134	*r* = 0.143; *p* = 0.252	*r* = 0.223; *p* = 0.072	*r* = 0.106; *p* = 0.397
**Bike Lumbar curv.**	*r* = − 0.011; *p* = 0.929	*r* = 0.115; *p* = 0.359	*r* = − 0.123; *p* = 0.325	*r* = 0.046; *p* = 0.716	*r* = − 0.150; *p* = 0.230	*r* = − 0.045; *p* = 0.722	***r* = − 0.284; *p* = 0.021**	*r* = 0.209; *p* = 0.092	*r* = 0.057; *p* = 0.648	*r* = 0.074; *p* = 0.553	*r* = 0.068; *p* = 0.586	*r* = 0.165; *p* = 0.187	*r* = − 0.198; *p* = 0.112	***r* = − 0.323; *p* = 0.008**
**Bike Pelvic tilt**	*r* = − 0.191; *p* = 0.125	***r* = − 0.333; *p* = 0.006**	*r* = 0.049; *p* = 0.696	***r* = − 0.251; *p* = 0.042**	*r* = 0.178; *p* = 0.153	*r* = − 0.124; *p* = 0.319	*r* = 0.204; *p* = 0.101	***r* = − 0.328; *p* = 0.007**	***r* = − 0.325; *p* = 0.008**	***r* = − 0.347; *p* = 0.004**	***r* = − 0.351; *p* = 0.004**	***r* = − 0.380; *p* = 0.002**	*r* = 0.102; *p* = 0.413	***r* = 0.385; *p* = 0.001**
**Surfboard Thoracic curv.**	***r* = 0.291; *p* = 0.019**	*r* = 0.107; *p* = 0.396	***r* = 0.298; *p* = 0.016**	*r* = 0.054; *p* = 0.667	*r* = − 0.121; *p* = 0.337	***r* = 0.406; *p* = 0.001**	***r* = 0.311; *p* = 0.012**	*r* = 0.185; *p* = 0.139	*r* = 0.206; *p* = 0.100	*r* = 0.117; *p* = 0.352	*r* = 0.170; *p* = 0.176	*r* = 0.038; *p* = 0.762	***r* = 0.289; *p* = 0.019**	***r* = 0.269; *p* = 0.030**
**Surfboard Lumbar curv.**	*r* = 0.167; *p* = 0.184	***r* = 0.511; *p* = 0.000**	*r* = − 0.228; *p* = 0.068	***r* = 0.400; *p* = 0.001**	*r* = − 0.224; *p* = 0.073	*r* = 0.079; *p* = 0.532	***r* = − 0.276; *p* = 0.026**	***r* = 0.351; *p* = 0.004**	***r* = 0.441; *p* = 0.000**	***r* = 0.447; *p* = 0.000**	***r* = 0.462; *p* = 0.000**	***r* = 0.356; *p* = 0.004**	*r* = 0.005; *p* = 0.971	***r* = − 0.258; *p* = 0.038**
**Surfboard Pelvic tilt**	*r* = 0.176; *p* = 0.16	*r* = 0.012; *p* = 0.927	*r* = 0.228; *p* = 0.067	*r* = 0.028; *p* = 0.825	*r* = 0.037; *p* = 0.767	*r* = 0.027; *p* = 0.829	***r* = 0.31; *p* = 0.012**	***r* = − 0.253; *p* = 0.042**	*r* = − 0.025; *p* = 0.845	*r* = 0.020; *p* = 0.877	*r* = − 0.004; *p* = 0.977	*r* = − 0.044; *p* = 0.725	*r* = 0.197; *p* = 0.117	*r* = 0.234; *p* = 0.061
**Air walker Thoracic curv.**	***r* = 0.311; *p* = 0.012**	*r* = 0.100; *p* = 0.426	***r* = 0.336; *p* = 0.006**	*r* = 0.025; *p* = 0.846	*r* = − 0.169; *p* = 0.179	***r* = 0.403; *p* = 0.001**	***r* = 0.321; *p* = 0.009**	*r* = 0.170; *p* = 0.176	*r* = 0.177; *p* = 0.157	*r* = 0.131; *p* = 0.3	*r* = 0.161; *p* = 0.200	*r* = 0.100; *p* = 0.43	***r* = 0.278; *p* = 0.025**	*r* = 0.210; *p* = 0.092
**Air walker Lumbar curv.**	*r* = − 0.115; *p* = 0.362	*r* = − 0.05; *p* = 0.693	*r* = − 0.109; *p* = 0.387	*r* = − 0.082; *p* = 0.518	*r* = − 0.065; *p* = 0.605	*r* = 0.053; *p* = 0.675	***r* = − 0.295; *p* = 0.017**	***r* = 0.346; *p* = 0.005**	*r* = − 0.03; *p* = 0.811	*r* = − 0.052; *p* = 0.679	*r* = − 0.042; *p* = 0.737	*r* = 0.056; *p* = 0.655	*r* = − 0.177; *p* = 0.158	*r* = − 0.217; *p* = 0.083
**Air walker Pelvic tilt**	*r* = 0.077; *p* = 0.544	*r* = − 0.045; *p* = 0.72	*r* = 0.139; *p* = 0.269	*r* = − 0.031; *p* = 0.809	*r* = 0.026; *p* = 0.835	*r* = − 0.031; *p* = 0.808	*r* = 0.207; *p* = 0.098	*r* = − 0.232; *p* = 0.063	*r* = − 0.025; *p* = 0.843	*r* = − 0.021; *p* = 0.87	*r* = − 0.024; *p* = 0.851	*r* = − 0.089; *p* = 0.481	*r* = 0.102; *p* = 0.417	*r* = 0.169; *p* = 0.177

**Notes.**

SBR Single bonny rider curv curvature FP Final Position IP Initial Position

According to the multiple linear regression analysis (Table 4), the variables height and the cormic index explained most of the spinal changes during the use of the different OFE. More specifically, the subjects with a higher cormic index were more at risk of increasing their thoracic kyphosis in the SBR, row, bike, surfboard and air walker. In turn, those subjects with a higher cormic index showed a higher risk of decreasing lumbar lordosis towards kyphotic positions in the IP of the SBR, while those subjects with a higher height had a higher risk of decreasing lumbar lordosis towards kyphotic positions in the FP of the SBR and row, bike and surfboard machines. Likewise, taller subjects had a greater risk of decreasing pelvic anteversion towards pelvic retroversion positions in SBR, row and bike.

**Table 4:** Stepwise multiple linear regression analysis of the relationship of differentiation of spinal curvatures in the outdoor fitness machines with anthropometric variables.

	**Difference** **OFE-standing**	**R** ^**2**^	***p*** **Value**	**Included independent variables**	**Standardized Coefficient** **(β)**
SBR	IP Thoracic curv. (°)	.060	.049	Cormic index	0.245
	IP Lumbar curv. (°)	.080	.023	Cormic index	0.283
	IP Pelvic tilt (°)	.160	.001	Height	−0.400
	FP Thoracic curv. (°)	.111	.007	Cormic index	0.332
	FP Lumbar curv. (°)	.172	.001	Height	0.415
	FP Pelvic tilt (°)	.163	.001	Height	−3.486
Row	IP Thoracic curv. (°)	.175	.001	Cormic index	0.419
	IP Lumbar curv. (°)	.089	.016	Height	0.298
	IP Pelvic tilt (°)	.197	.000	Height	−0.444
	FP Lumbar curv. (°)	.152	.001	Height	0.390
	FP Pelvic tilt (°)	.114	.006	Height	−0.338
Bike	Thoracic curv. (°)	.158	.001	Cormic index	0.398
	Lumbar curv. (°)	.100	.010	Height	0.316
	Pelvic tilt (°)	.137	.002	Height	−0.370
Surfboard	Thoracic curv. (°)	.127	.004	Cormic index	0.357
	Lumbar curv. (°)	.368	.000	Height	0.606
Air walker	Thoracic curv. (°)	.088	.016	Cormic index	0.297

**Notes.**

OFE outdoor fitness equipment SBR Single bonny rider curv curvature FP Final Position IP Initial Position

## Discussion

Most elderly people suffer from changes in the sagittal spine disposition in different positions, due to a loss of strength, muscle shortening, reduced mobility, and stiffness of passive structures such as ligaments and discs (Norasteh et al., 2019). Therefore, it is necessary to evaluate the spine disposition not only in standing, but also in different positions in daily life and sports practice in order to analyze the adaptations that occur during these activities (Abelleira-Lamela et al., 2020; López-Miñarro, García & Medina, 2009). However, most previous studies have only analyzed middle-aged and older adults in a standing position (Asai et al., 2017; Elsayed et al., 2018; Kasukawa et al., 2017; Keller et al., 2005; Ohyama et al., 2019). For this reason, in the present study, we analyzed the sagittal disposition of the spine of middle-aged and older adults in standing position, but also in the different OFE that are most frequently installed in the bio-healthy parks.

The main finding of the present investigation was that in general, there were significant differences in the sagittal spine disposition and pelvic tilt during the execution of the exercises with the machines, as compared to the relaxed standing position in middle-age and older adults.

In the OFE that were performed in the two-legged position, as was the case with the surfboard and air walker, an increase in thoracic kyphosis was found when using these OFE, without changes in lumbar curvature or pelvic tilt in general. Coinciding with these results, previous studies have shown that when a frontal grip has to be performed, with internal rotation and/or shoulder adduction, it is common to find greater thoracic kyphosis than in a relaxed standing position (Abelleira-Lamela et al., 2020; López-Miñarro, Rodríguez-García & Santonja Medina, 2010; Muyor, 2015), especially in the event that this provision is accompanied by a maintained grip (Imagama et al., 2014; Kordi-Yoosefinejad et al., 2019). This is especially relevant considering that the presence of hyperkyphotic thoracic curvatures during the execution of fitness exercises could lead to a lower involvement of the musculature that is mainly involved in the exercise. This could result in a greater shortening of the flexor and internal rotator muscles in the shoulder, a decrease in joint mobility, and changes in biomechanics and in the position of the center of gravity, among other factors (Imagama et al., 2014; Kordi-Yoosefinejad et al., 2019), which could can increase the risk of falls, back pain and loss of function (Norasteh et al., 2019).

In the sedentary machines, the grip was in a more neutral position and with less activation of the upper limb musculature, which could influence the degree of tension of the stabilizing musculature in the scapulo-humeral joint and the thoracic area to adopt more neutral positions (Imagama et al., 2014; Kordi-Yoosefinejad et al., 2019). However, in sedentary exercises performed in OFE, there was a decrease in lumbar lordosis and pelvic anteversion. Previous studies found the same tendency toward decreased lumbar lordosis and pelvic anteversion when analyzing the disposition of the spine and pelvic tilt during sedentary exercise positions in older individuals (Norasteh et al., 2019). This disposition was also found during other sports actions that required executing force from a sitting position (Abelleira-Lamela et al., 2020; López-Miñarro, Rodríguez-García & Santonja Medina, 2010; Muyor, 2015). More specifically, previous studies found that in the case of starting from a sedentary, sitting position, the technical gesture requires performing a pulling movement with the upper limbs fully extended, the trunk inclined and the knees semi-extended, as is the case of row and SBR, there was an adaptation in the pelvic tilt and lumbar disposition (Abelleira-Lamela et al., 2020; López-Miñarro, Muyor & Alacid, 2012). This is especially relevant if we take into account that people who adopt a kyphotic position in the lumbar area in situations of load mobilization have a higher probability of suffering pathologies such as herniated and protruded discs, low back pain, spondylolysis and spondylolisthesis, among others (McGill, Cannon & Andersen, 2014). Therefore, it would be advisable to modify the design of this type of machines to allow for a normal disposition of the lumbar spine during the execution of guided exercises (McGill, Cannon & Andersen, 2014). Despite these promising results, questions still remain.

Another of the objectives of the present investigation was to analyze whether anthropometric variables could condition the sagittal disposition of the spine and pelvic tilt during the use of the OFE, finding that anthropometric variables significantly influenced the adaptations in the sagittal spine disposition and pelvic tilt. Previous studies have already pointed out that anthropometric variables could condition the sagittal spine disposition when performing actions involving the application of force (Abelleira-Lamela et al., 2020; Elsayed et al., 2018). However, until now, no studies had been conducted which analyzed this issue in OFE.

More specifically, it was found that in general, the users with a greater height and/or with a higher cormic index, whose sitting height was proportionally larger than the sitting height, were more likely to be taller (Esparza-Ros, Vaquero-Cristóbal & Marfell-Jones, 2019), have a greater probability of presenting kyphotic thoracic curves, as well as kyphotic positions of the lumbar area, together with a greater probability of showing pelvic retroversion in the machines that were executed in a sedentary position. In addition, in the ANCOVA analysis, height was found to be a covariate that significantly influenced pelvic tilt in the OFE performed in sitting in the IP and FP, as well as thoracic curvature in the surfboard. The differences in pelvic tilt may have been due to the participants were sitting on the OFE and the taller subject will be able to perform the grips in a more upright position, thus requiring less pelvic tilt in terms of grip as it has been found using other kinds of sport equipment (Abelleira-Lamela et al., 2020; López-Miñarro, Muyor & Alacid, 2012; Muyor, 2015). Regarding thoracic curvature, it was found that height influenced the surfboard, but not the air walker, even though both were in standing position. This could be due to the grip, since the air walker has a higher frontal grip, while the surfboard has a lateral grip at hip height, so that a greater trunk flexion is required to maintain the grip. Previous studies have already found that the number of subjects with thoracic hyperkyphosis is higher in this type of position when there is no proper postural hygiene (Farrag et al., 2015; Mawston, McNair & Boocock, 2007).

In contrast, the anthropometric parameters related to the transverse dimension had no influence on the sagittal disposition of the spine in middle-aged or older adults. This could be a consequence of the fact that one of the problems presented by the OFE in relation to ergonomics is that it cannot be adjusted according to the anthropometric characteristics of the users (Chow & Wu, 2019), in contrast to what happens in the classic fitness room equipment (Chow & Wu, 2019). Thus, it is likely that in those subjects who are taller or whose trunk is longer, the grip remains low, having to perform a thoracic hyperkyphosis in order to hold themselves. The disposition of the thoracic spine could in turn condition the disposition of the lumbar curvature and pelvic tilt (López-Miñarro et al., 2017; Vaquero-Cristóbal et al., 2015), which would explain the presence of inversions in the lumbar area and pelvic retroversion positions accompanying thoracic hyperkyphosis dispositions, especially when the execution has to be carried out from a sedentary position (López-Miñarro et al., 2017; Vaquero-Cristóbal et al., 2015). This tendency towards flattening of the lumbar curvature and pelvic tilt observed in the sagittal spine disposition during the use of OFE would be contrary to the ideal position. In this regard, the curved disposition of the spine in the sagittal plane is essential, as the strength of an element is equal to the number of curvatures squared plus one (Kapandji, 1981), the almost aligned disposition of the lumbar spine and the pelvic tilt would detract from the strength of the spine. In addition, the spine is more resistant to axial compression and more stable with the presence of these curves than without them (Panjabi et al., 1985), allowing for an efficient absorption of the loads applied to the spine (Adams & Dolan, 1996; Kapandji, 1981) and increasing the efficiency of the paravertebral musculature (Dickson, 2004). Therefore, the misalignments of the thoracic and lumbar curvatures, together with the external load caused by the performance of the exercises, could increase intervertebral stress and spinal loads (Keller et al., 2005). For all these reasons, it would be necessary to redesign the OFE, evolving towards models that would allow adjusting the distances between footrests, seats and grips according to the size and seated size of middle-aged and older adults.

Another of the objectives of the present investigation was to analyze whether the sex covariate influenced the changes that occurred in the sagittal spine disposition on the OFE. A much debated question is whether sex influence sagittal spine disposition. Specifically, some papers have found that women show a greater probability of presenting lumbar hyperlordosis than men in adulthood (Bailey et al., 2016), while others have showed similarity in the sagittal spine disposition between men and women (Endo et al., 2012; Janssen et al., 2009; Yeh et al., 2018). In the present investigation it was found that sex did not influence the sagittal spine disposition and pelvic tilt adopted in the OFE. This could be due to the fact that other factors are affecting the adaptations that occur in the sagittal arrangement of the spine when using this type of fitness equipment.

As a strength of the research, this is the first study that analyzes the disposition of the spine in the OFE, and the sample investigated was large. Regarding the limitations of the present study, although the sample of the present study was large as compared to other, similar studies, the sample of men was somewhat reduced, perhaps because men do not find these types of machines useful and, therefore, this is another reason to investigate the safety and effectiveness of the OFE. In addition, since we only had one model of each machine and not different prototypes of each one, we could not analyze the influence of changes in the disposition of the machines on the sagittal spine disposition. Thus, this could be a future line of research with which to analyze whether the modification of the OFE allows for a safer and healthier sagittal spine disposition and pelvic tilt of its users. On the other hand, it will be possible to contrast the results of the present investigation in professional equipment that does allow an adaptation of the equipment according to the anthropometric characteristics of the user, such as fitness equipment as well as looking for new ways of using the machines to make them as versatile as possible. Another limitation of the present investigation was that the evolution of low back pain or other spinal pathologies during the use of OFE was not analyzed. This should be addressed in future research.

## Conclusions

Middle-aged and older adults show spinal misalignments when using the OFE with respect to the standing position, showing a significant decrease in the thoracic kyphosis in the IP of SBR and ROW, and a significant increase in the surfboard and air walker, and in the FP of Row; a significant decrease in the lumbar lordosis in all OFE with the exception of the air walker; and a significant decrease in the pelvic anteversion in all the OFE in sitting (SBR, Bike and Row). Height and the cormic index conditioned these adaptations, showing that middle-aged and older-adults with higher height and cormic index had a higher probability of showing increased thoracic kyphosis, lumbar kyphosis, and pelvic retroversion. This are the reason for the recommendation of the manufacturing of safer outdoor fitness equipment that allows users to adjust them to their anthropometric needs.

## Supplemental Information

10.7717/peerj.12657/supp-1 Supplemental Information 1 Full database

10.7717/peerj.12657/supp-2 Supplemental Information 2 Codebook of the database for categorical data

## Additional Information and Declarations

### Data Availability

The following information was supplied regarding data availability: The database is available in the Supplementary File.
