# Supplementary material for: Sagittal spine disposition and pelvic tilt during outdoor fitness equipment use and their associations with kinanthropometry proportions in middle-aged and older adults
Source: PeerJ. 2021 Dec 20;9:e12657. doi: 10.7717/peerj.12657 (PMC8697770; doi:10.7717/peerj.12657)
Supplement: Supplemental Information 2 [file peerj-09-12657-s002.docx]

**Codebook of the database for categorical data**

Sex: 1=Men; 2=Women
